# Supplementary material for: Complex‐centric proteome profiling by SEC‐SWATH‐MS
Source: Mol Syst Biol. 2019 Jan 14;15(1):e8438. doi: 10.15252/msb.20188438 (PMC6346213; doi:10.15252/msb.20188438)
Supplement: Supplementary file 8 — Dataset EV7 [file MSB-15-e8438-s008.zip › feature_plots_string/Q14493.pdf]

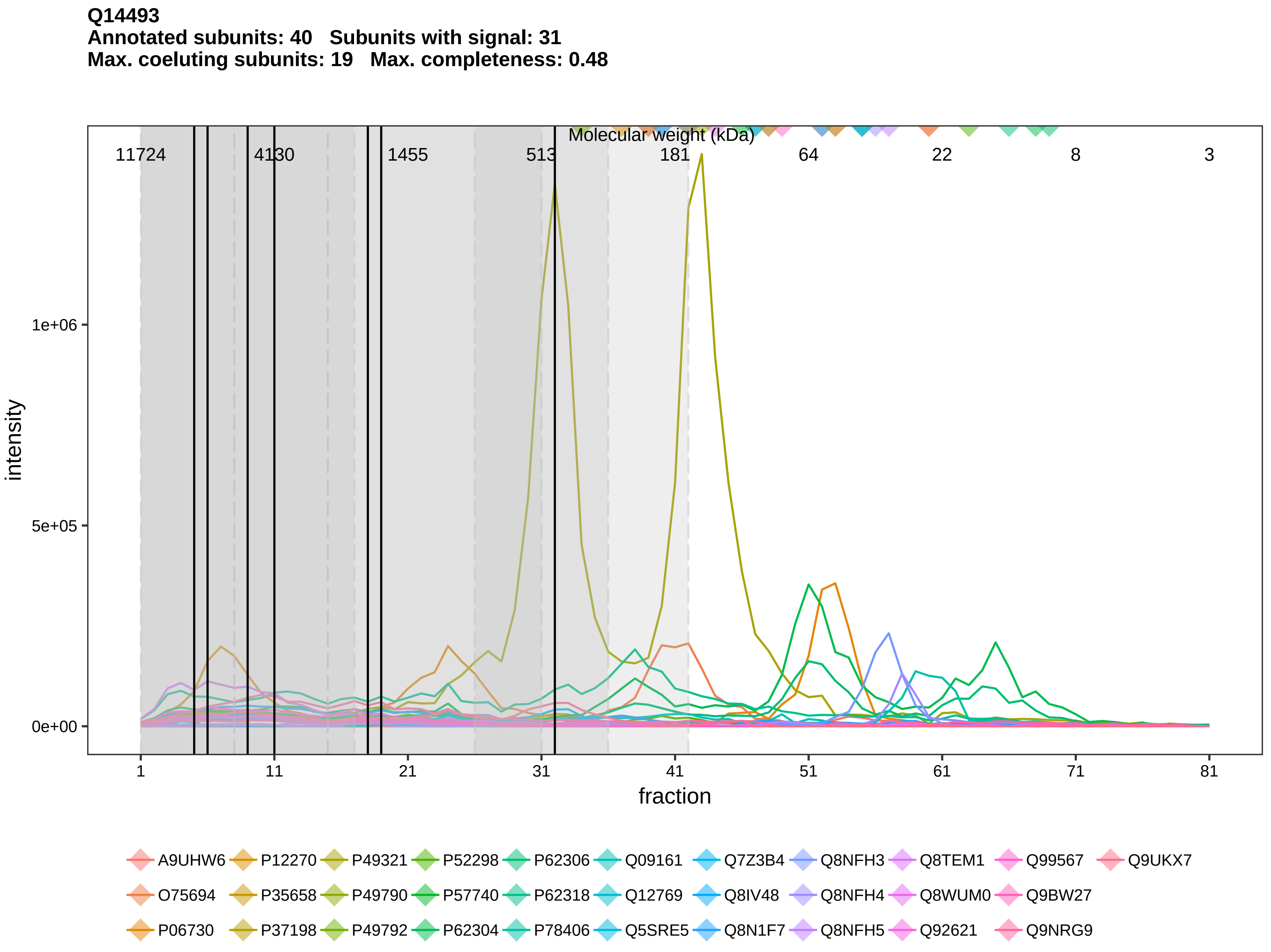

Q14493
Annotated subunits: 40 Subunits with signal: 31
Max. coeluting subunits: 19 Max. completeness: 0.48
Molecular weight (kDa)
11724
4130
1455
513
181
64
22
8
3
intensity
1e+06
5e+05
0e+00
fraction
1
11
21
31
41
51
61
71
81
A9UHW6
P12270
P49321
P52298
P62306
Q09161
Q7Z3B4
Q8NFH3
Q8TEM1
Q99567
Q9UKX7
O75694
P35658
P49790
P57740
P62318
Q12769
Q8IV48
Q8NFH4
Q8WUM0
Q9BW27
P06730
P37198
P49792
P62304
P78406
Q5SRE5
Q8N1F7
Q8NFH5
Q92621
Q9NRG9
